# The Role of Social Determinants of Health in Promoting Health Equality: A Narrative Review

**DOI:** 10.7759/cureus.33425

**Published:** 2023-01-05

**Authors:** Khushbu Chelak, Swarupa Chakole

**Affiliations:** 1 Public Health and Epidemiology, Jawaharlal Nehru Medical College, Datta Meghe Institute of Medical Sciences, Wardha, IND; 2 Community Medicine, Jawaharlal Nehru Medical College, Datta Meghe Institute of Medical Sciences, Wardha, IND

**Keywords:** health services, health equality, health policies, social factors, social movement, health inequities, social determinants

## Abstract

Significant health disparities exist locally and even throughout the nation. Dipping health inequalities necessitates a focus on the inadequate spread of power, money, and resources, as well as the situations of daily living, which may be addressed through social determinants of health. This study aimed to review the role of health-related social factors in overcoming health disparities. We conducted a search of English-language literature, including studies published on health and health equalities or inequalities. Most reports show that social determinants of health have a higher effect on health. The elimination process of these health inequities occurs through well-designed economic and social policies. Every aspect of social determinants influences the health aspects of people; hence, some areas to focus on include employment, education, socioeconomic status, social support networks, health policies, and healthcare access. Launching interventions to reduce health disparities can help improve the community’s health and health equality.

## Introduction and background

According to the World Health Organization (WHO), social determinants of health (SDH) are defined as the circumstances in which humans are born, develop, live, earn, and age. At the international, regional, and state or local levels, the distribution of money, power, and resources shapes these circumstances [1]. The WHO Commission on Social determinants of Health (CSDH) has stated that progress on SDH is the most successful means of enhancing all people’s well-being and raising disparities [2]. The WHO established the CSDH based on SDH intervention, which is the most effective strategy to improve well-being and reduce inequality [2]. Important aspects include governmental, financial, and traditional organizations, based on factors such as manageable healthcare and learning organizations; safe ecological conditions; aesthetically pleasant neighborhoods; and the availability of nutritious food [3]. Nowadays, health challenges such as being overweight, cardiovascular diseases, diabetes, and depression are prominent, wreaking havoc upon people because of the increasing demands of a high lifestyle. This leads to people suffering from non-communicable diseases. These socioeconomic variables contribute to societal stratification and health disparities among persons of different social and economic classes, genders, and ethnicity.

History of social determinants of health inequality

In the 19th century, people started becoming aware of the factors that had an impact on the health of the population [4]. Rudolf Virchow, a pioneer in this field, testified on the role of poverty in generating a disease that led to a plague outbreak in Prussia [4]. Friedrich Engels also studied to find out about the increased mortality. After that, Salvador Allende tried in Chile to demonstrate the importance of political and social variables in people’s health inequities [4]. All of them tried to frame how factors influence health and what role they play. Marmot emphasized that the workplace may be an important location for addressing disparities. Similarly, changing housing might have an impact on physical and mental health [5]. Cutting across the structural inequalities, health inequality is a more contemporary challenge and possibly a consequence of the imbalances in development planning and economic design [6]. Interventions on health and its disparities help overcome further problems [7]. There is a long history of housing evidence from several reviews [8]. People suffering from the financial crisis and economic disparities were also among the many who were deeply affected by the growing socioeconomic demands in the early days.

## Review

Methodology

**Figure 1:**
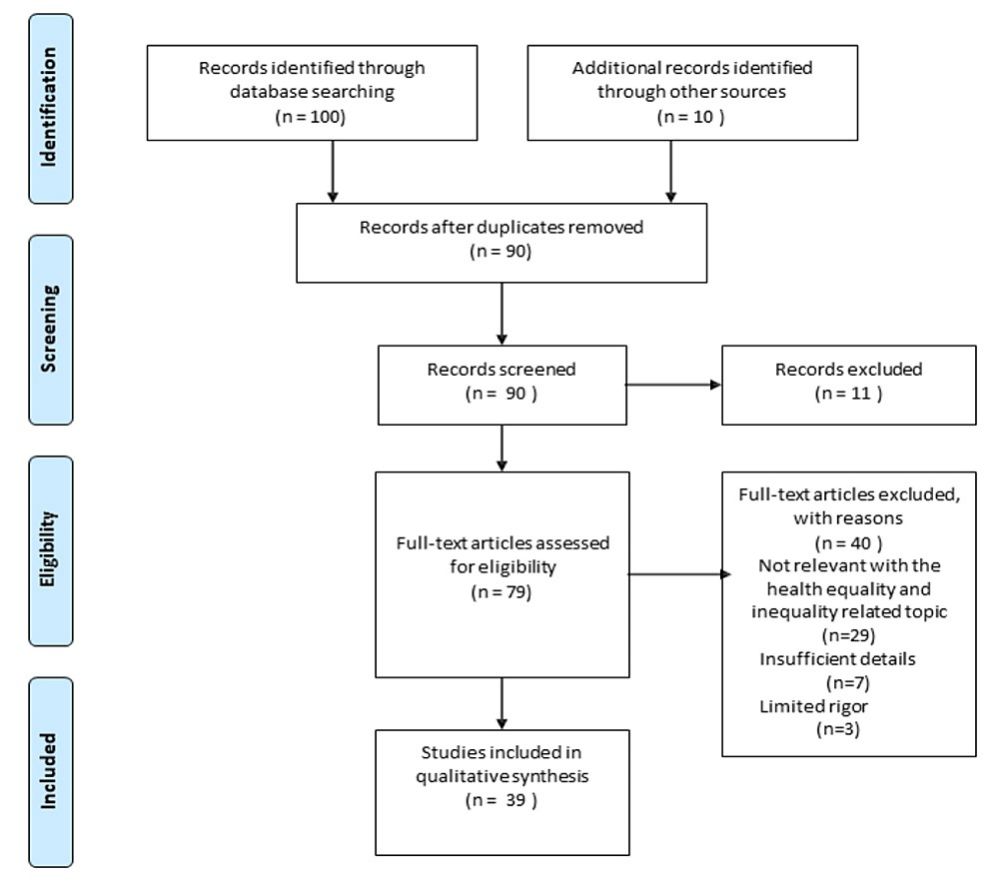
Inclusion and exclusion criteria of this study.

This article presents a narrative review of SDH in promoting health equality. PubMed and Google Scholar were used to find all original and review articles with original reports. A set of keywords and Medical Subject Headings (MeSH) terms related to health inequalities and SDH were used. Keywords used were social inequalities, social inequities, poverty, health determinants, behavior, economic status, and social movement. The following MeSH terms were used interchangeably and in combination to find all relevant articles: social determinants, health inequities, and social movement. All free full-text PubMed Central articles were searched using Pubmed and Google Scholar. Studies that discussed the relationship between health inequities, the importance of social determinants, health inequities, health policies, social factors, health equality, and social movement were included. Articles that reviewed SDH in a more general way and whose main focus was not health inequalities and equalities were excluded (Figure 1).

Social determinants of health

**Figure 2:**
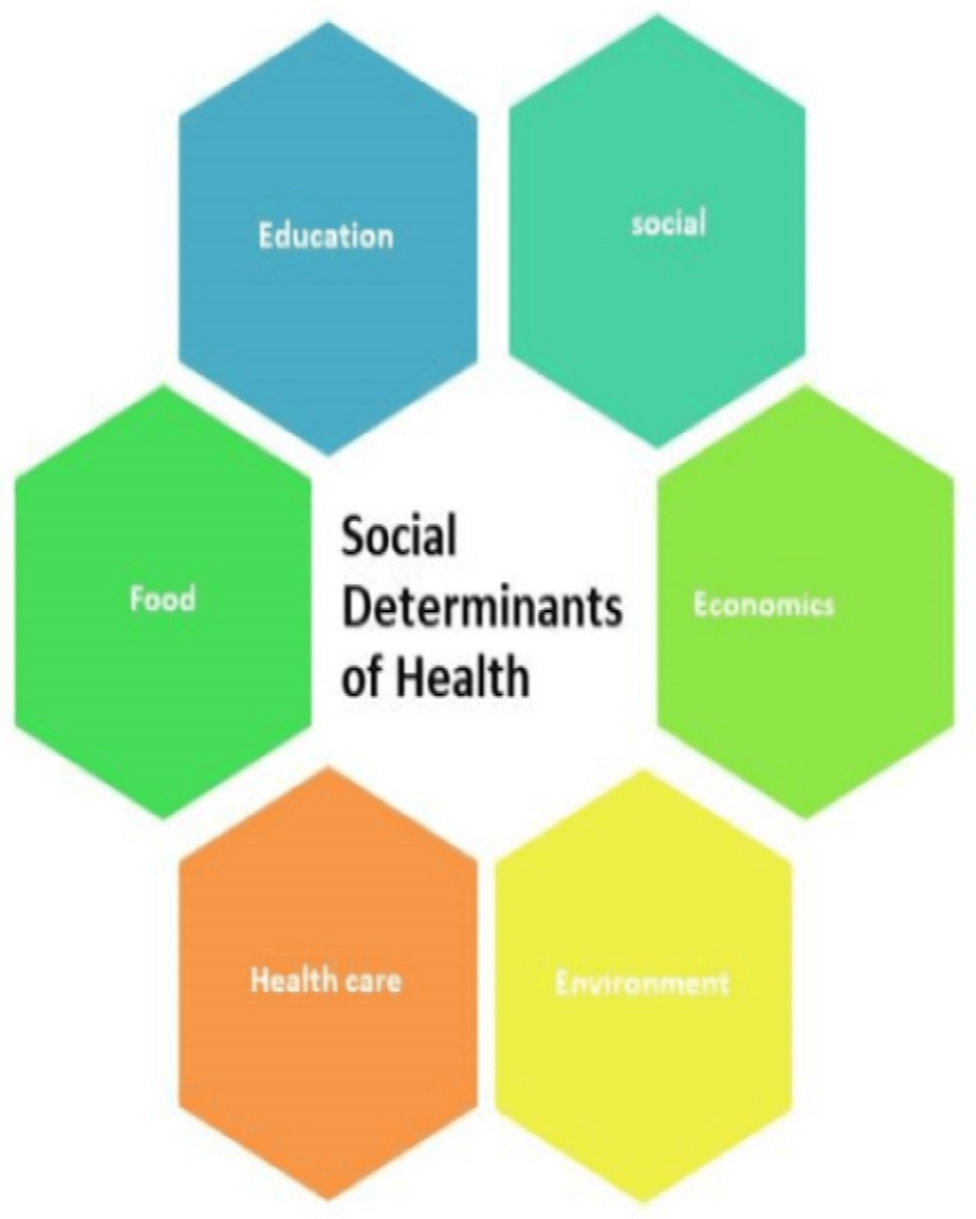
Social determinants of health Source: Open access journal under a CC-BY license contributed by social determinants of health. Available at: https://www.who.int/health-topics/social-determinants-of-health#tab=tab_1 [9].

A subcategory of health factors is SDH, as shown in Figure 2.

The most significant health factors include government policy, medical availability, individual behavioral choices, and biological and genetic features [3]. Examples of SDH include occupation, job status, workplace safety, level of income, opportunities for education, job and place of work protection, inequity between men and women, and segregation based on race. The various health aspects of SDH include food poverty and limitations of access to nutritious food options, housing, and helpful facilities available; early childhood growth and experiences; inclusion in the community and social assistance; the prevalence of crime and exposure to violent behavior; neighborhood circumstances and physical environment; and possibilities for recreation and leisure, as shown in Table 1.

**Table 1:** Various aspects of social determinants of health. Source: Open access journal under a CC-BY license contributed by social determinants of health (2018). Available at: https://www.christenseninstitute.org/wp-content/uploads/2018/10/Social-Determinants-of-Health-Table.png [10].

Economic stability	Necessities	Demographics and social context	Environmental
Employment status	Safe, secure, quality housing	Gender identity/inequality	Crime rate/violence
Income level	Access to affordable, healthy food options	Sexual orientation/discrimination	Access to transportation
Health insurance status	Access to clean drinking water	Ethnicity/racism	Safety of the built environment
Expenses	Air quality	Cultural identity	Parks, green space
Financial safety net	Utilities (heat, etc.)	Language barrier	Recreational and leisure opportunities
		Immigration status	Availability of healthcare
		Social network, capital, and support	

Social determinants of health indicators

Social determinants determine how health is affected, how they play a significant role in influencing health, and how we can improve health for all. Some of the effects of social determinants affect health in the long term. For example, a less educated person might have less knowledge about how to utilize resources which may affect their ability to use resources to the fullest. Thus, social determinants play a role which must be recognized and improved.

Socioeconomic Status

Financial stress and socioeconomic status are a combination of a family’s or ordinary citizen’s profession, academic performance, wealth, and economic standing. Wealth and power are characteristics that influence a person’s socioeconomic status. The total amount earned in earnings or compensation over a year is income. Income limits a family’s overall lifestyle and influences their consumption habits [11]. Discrimination based on caste, creed, and gender makes a person vulnerable, enabling them to stop asking for more. They are subjugated to extreme pressure, which only worsens their mental status and health. Health education and programs must be used to educate people about how beneficial it is to count everyone as a whole. The promotion of health equality and equity remains the most significant goal of balancing financial stress.

Equality Education

Education is a means of improving one’s socioeconomic standing. A wealthy family’s socioeconomic status suggests a higher chance of enrolling and graduating from college. Family background, rather than other factors, such as supplemental educational services, significantly influence how much and what kind of education people get and what kind of employment they obtain [11]. The goal of improving income equality and eradicating poverty through education has not been met. Higher wages and social policies that support low-income families are needed to enhance students’ social and economic conditions [11]. Educational perspectives allow us to take a comprehensive and clearer view of the causes of health and disease in a population and must be paid attention to [4].

Gender Inequality and Age Inequality

Women earn less and are more likely to be poor. Institutional discrimination in a patriarchal society, where women were supposed to be mothers and spouses rather than part of the formal workforce, is to blame for gender inequality [11]. Poverty is more prevalent among the youngest and oldest population groups. Children are more likely to be poor than other age groups. School attainment, high school graduation rates, and reading skills are all impacted by poverty [11]. Health inequality does not mean just some kind of health difference but the differences in health like that of a pregnant woman who has fewer resources and is deficient and the newborn child who might be underweight leading to problems such as stunting and growth retardation. This adversely affects the opportunities and performances of those afflicted by it and can be corrected by successfully evaluating the determinants affecting health [6].

Economic Inequality

The United States has the least poverty rates and the most restrictive social policies regarding escaping poverty. Except for Mexico and Turkey, all other developed countries have more significant income disparity than the United States. Young people, full-time workers in low-status jobs, people of color, illiterate people, and women are most likely to be poor [11]. In influenced market knowledge and customer needs individualized society, incomes or wealth are alternate socioeconomic indicators [3].

Economic Power

Economic power is the ability to improve the standard of living of a country or business. Economic power represents the status of people with higher socioeconomic status wielding more power than those with lower socioeconomic status. For example, employment provides income that shapes choices about housing, child care, education, medical care, and food, among others. Influence is a factor of being able to produce, buy, and sell. Power is the primary force in today’s era. Because the curriculum is governed by teachers, school board members, and national standards, teachers and students have power connections [11].

Implications of a System Approach

In a systems approach, the current state of affairs and its factors are both causes and outcomes. Rather than a linear path of socioeconomic variables leading to various health outcomes, they are interconnected in a causal web. The feedback loops result in outcomes that influence the causes. Low income and deprivation, for example, lead to inferior health outcomes, exacerbating the group’s poor and worsening health. A more advanced model, such as the system dynamic model, is necessary to operationalize [12]. Early events and life cycle, occupational considerations, social ties (social networks and support, discrimination, neighborhood characteristics), and healthcare are all identified as social risk factors [13]. Eliminating these health inequities indicates that well-designed economic and social policies can promote health and health equity. It outlines 10 guidelines to keep in mind while launching interventions to reduce health disparities [14]. The circumstances increase their impact on life [15]. The physical atmosphere, opportunities for learning, suitable housing, occupation, and wealth are examples of these circumstances, known as SDH [16]. Recommendations such as improving living conditions and inequalities among people are justified in their own right but the way these are linked to health is problematic [17]. The magnitude of inequalities should be viewed with caution because the study does not take caste into account, potentially exaggerating socioeconomic inequalities [18].

Policies for Improving Health Equality

While numerous public policies contribute to public health and equality, enhancing public health is not the society’s or the government’s only goal. Although these initiatives have been effective at commencing actions addressing SDH, continuing inequities, and diverse social, economic, and cultural differences across India, more cooperation is required across the current programs of different ministries [18], resulting in policy incoherence may develop. Due to a lack of policy coherence throughout the government, one branch of the government may ensure the introduction of a national development plan of action, for example, TB free response to the change of the WHO [19]. At the same time, other parts promote exports, industrialization, and proposals that are dangerous to human life. The single cause for these discrepancies is a lack of knowledge among areas of the connections between health and quality of life, on the one hand, and more significant health determinants, such as productivity expansion, on the other. Another cause is that unrelated initiatives may have unexpected consequences that are not monitored or addressed. In its preamble, the Indian Constitution provides core values for establishing a social order in the country. An orderly society is built on these core values. Equality, various freedoms, socioeconomic justice, and individual dignity are fundamental principles for governing a democratic country like India. The policy approach will protect the social rights of people [20].

The healthcare system must comprehend the obligations of other parts and establish mutual consideration of health, its consequences, and great social welfare or life characteristics to contribute to policy coherence across government. It needs novel solutions and institutions that create avenues for debate and decision-making that cut across typical policy silos in government. In practice, this entails taking a variety of acts, such as facilitating seminars of government policymakers, program leaders, and healthcare provider organizations, to promote policy, service, and program coherence in response to the needs of disadvantaged groups, such as via conferences conducted at numerous organizational stages and with private and government providers. For evaluation of policy progress and pitfalls, from a theoretical perspective, several policy-making frameworks can be used to describe how programs are developed and executed [21].

The policy windows model by Kingdom (1995) is crucial as it illustrates how and why issues become part of the policy agenda before implementation [22]. Three streams are coupled or decoupled problem, policy, and politics; according to Kingdom, open and close strategy opportunities. The gathering of proof regarding health inequality is essential but not enough for policy change. Problems must be viewed or identified as problems that can be addressed by legislation. Complicated by the fact that general populace initiatives in largely unrelated domains may have population health implications [23]. The collection of facts, particularly the Acheson Report, has aided in the designation of health disparities as a political issue. Similar inquiries have formed the issue in other places [24].

Socioeconomic Determinants and Health Inequities

SDH must all be incorporated into public health services to reduce health disparities. Health services must be adapted to the demands of distinct population groups. Due to the build-up of difficulty through several areas and over the life course, different social groups in the population differ in their empowerment to participate in health interventions. Many public health programs have not met or are not meeting their health equity targets due to a lack of healthcare-specific interventions and a failure to reach out to vulnerable people and address significant social variables that affect public health. Disparities exist between public and private health systems [25]. Policy efforts at the health system level are required to monitor and improve these disparities [25]. The coronavirus disease 2019 (COVID-19) pandemic has had the greatest impact on groups that have faced discrimination and historical injustices [26]. Poor living conditions and exploitative labor have become more prevalent, allowing for inequitable income distribution and health risks [26]. Governments have exploited the pandemic to further erode civil and human rights and promote extractives [26]. A post-COVID-19 world must ensure equity, social justice, solidarity, and a shift in the balance of power and resources for poor and marginalized people [26].

Lower-income societies with lower smoking rates have a lower incidence of lung cancer [27]. Individual smoking patterns or different rates of illness prevalence and incidence among social groups, i.e., inequalities, are caused by balances or imbalances in community norms and social structures. Sick people are diametrically opposed to the overall healthy population [27]. The term health inequalities in SDH (SDHI) has recently been taken to refer to settings, social structures, social norms, and some determinants. Three primary paths have been proposed to describe how the social environment causes fitness inequities [28]. Social choice, or mobility of community, suggests that health, relatively more than the other way around, determines socioeconomic status. As a result, healthier people will be happier. They move toward a higher socioeconomic status than others who were less beneficial, resulting in inequities. Social causation claims that discrepancies in health outcomes are caused by a variety of unequally distributed material, psychosocial, and behavioral factors [29,30].

A life path viewpoint indicates various features throughout life (e.g., malnutrition in the maternal prenatal period, low learning services in infancy, physically hazardous employment, influence, and manifest illness trends across time). The eco-social method tries to assimilate these organic, communal, and natural variables in illness through a vigorous process of incorporation which means we accurately integrate natural effects from the substantial and the social world [7,31]. Over the last 40 years, research on health inequalities and growth has shed light on the income well-being trend [32]. Measuring the disparity between subgroups requires using different health data based on the relevant dimension of inequality (i.e., demographic, socioeconomic, or geographic factors) [33]. Monitoring health inequality at the national level assists in assessing the impact of policies, programs, and practices on the disadvantaged subgroup [33]. This priority will be given to the proposed Sustainable Development Goals. which ask countries to increase the income, gender, geographical location, race, age, ethnicity, disability, migrant status, and other relevant characteristics at the national level [34].

Conceptual Limitations of Inequalities

SDHI covertly and overtly embraces substantial parts of a Newtonian view of reality (i.e., reductionism, linearity, and hierarchy), as do most notions connected to health outcomes [35]. This reductive approach is represented by a factor influencing health outcomes, for example, socioeconomic stratification of mortality due to asthma and the selection of interventions that focus on a single determinant, for example, improving thermal comfort in homes with insufficient heat [36]. Another common assumption in this debate is linearity, which argues that determinants of inequalities can be used in a variety of situations [30]. Differential access to healthcare or education is presumed to be health disparities in results [37], essentially in a linear pattern, whether overtly or implicitly [38]. In the case of what works in terms of tackling health inequalities, disappointingly very less relevant reviews have been conducted [39].

## Conclusions

After reviewing the current literature on SDH and health inequalities, we conclude that economic and social factors such as poverty, social exclusion, and others are usually regarded as SDH. Interventions are the most effective strategies to improve everyone’s well-being and reduce inequalities. The severity of employment, geography, and education imply that better healthcare management and expanded education and work prospects are required. Additional efforts in this area will likely help overcome social health inequalities in communities and achieve health equality. Policies that reduce social disadvantage can reduce health inequalities. The current state of the health sector, for which the union and state governments are equally responsible, and the right to health is not equally distributed can only be corrected if the union and state governments start practicing and introducing more efforts to achieve health equality. Health rights should be given to all people, encouraging them to use more services. Hence, making them healthier, more productive, and fit.
